# A new, fast method to search for morphological convergence with shape data

**DOI:** 10.1371/journal.pone.0226949

**Published:** 2019-12-27

**Authors:** Silvia Castiglione, Carmela Serio, Davide Tamagnini, Marina Melchionna, Alessandro Mondanaro, Mirko Di Febbraro, Antonio Profico, Paolo Piras, Filippo Barattolo, Pasquale Raia

**Affiliations:** 1 Dipartimento di Scienze della Terra, dell’Ambiente e delle Risorse, University of Naples Federico II, Napoli, Italy; 2 Dipartimento di Biologia Ambientale, Sapienza Università di Roma, Rome, Italy; 3 Dipartimento di Scienze della Terra, University of Florence, Firenze, Italy; 4 Dipartimento di Bioscienze e Territorio, University of Molise, Pesche, Isernia, Italy; 5 Dipartimento di Ingegneria Strutturale e Geotecnica, Sapienza Università di Roma, Rome, Italy; 6 Dipartimento di Scienze Cardiovascolari, Respiratorie, Nefrologiche, Anestesiologiche e Geriatriche, Sapienza Università di Roma, Rome, Italy; Monash University, AUSTRALIA

## Abstract

Morphological convergence is an intensely studied macroevolutionary phenomenon. It refers to the morphological resemblance between phylogenetically distant taxa. Currently available methods to explore evolutionary convergence either: rely on the analysis of the phenotypic resemblance between sister clades as compared to their ancestor, fit different evolutionary regimes to different parts of the tree to see whether the same regime explains phenotypic evolution in phylogenetically distant clades, or assess deviations from the congruence between phylogenetic and phenotypic distances. We introduce a new test for morphological convergence working directly with non-ultrametric (i.e. paleontological) as well as ultrametric phylogenies and multivariate data. The method (developed as the function *search*.*conv* within the R package RRphylo) tests whether unrelated clades are morphologically more similar to each other than expected by their phylogenetic distance. It additionally permits using known phenotypes as the most recent common ancestors of clades, taking full advantage of fossil information. We assessed the power of *search*.*conv* and the incidence of false positives by means of simulations, and then applied it to three well-known and long-discussed cases of (purported) morphological convergence: the evolution of grazing adaptation in the mandible of ungulates with high-crowned molars, the evolution of mandibular shape in sabertooth cats, and the evolution of discrete ecomorphs among anoles of Caribbean islands. The *search*.*conv* method was found to be powerful, correctly identifying simulated cases of convergent morphological evolution in 95% of the cases. Type I error rate is as low as 4–6%. We found *search*.*conv* is some three orders of magnitude faster than a competing method for testing convergence.

## Introduction

A species’ phenotype depends on its ancestral state and the responses to selection or drift it experiences since that state. Selection towards similar optima in different parts of a tree (which could be described by the Ornstein–Uhlenbeck (OU) mode of evolution [1,2]) generates a pattern of evolutionary convergence [3–6]. Convergence is an intensely studied macroevolutionary pattern [7–11]. Evolutionary convergence is often invoked to explain instances of morphological resemblance between phylogenetically distant clades. Well-known examples include the evolution of wings in bats and birds [12,13], neck elongation in sauropods and giraffes [14], bone cracking ability in percrocutids, borophagine canids and hyaenids [15], hypsodont molars in grazing ‘ungulates’ [16,17], or the repeated occupation of specific ecomorphs by unrelated *Anolis* species in different Caribbean islands [18,19]. Examples of repeated convergence within a clade, known as iterative evolution, include the evolution of trenchant-heeled lower molar talonids in several canid lineages [12] and elongated and laterally-compressed upper canines within the cat family, Felidae [20]. This by no means exhaustive list just represents a brief account of a diffuse, widely occurring evolutionary pattern [21].

Current methods to address patterns of morphological convergence [22] rely on either: i) the phenotypic analysis of groups of species falling in some pre-selected state (i.e. qualitative categorization) as compared to their ancestors [23]; ii) fitting several OU models to different clades in the phylogenetic tree to see if they evolve towards the same peak (i.e. whether distant clades can be statistically collapsed under a common evolutionary regime [24]); iii) assessing the congruence between phylogenetic and phenotypic distances [18,25] or iv) studying the trajectory of phenotypic change across multiple evolutionary levels [26]. All these methods have advantages and shortcomings. For instance, the comparison of phenotypic to phylogenetic distance matrices may reveal departures from the expected association between the two for reasons other than convergence [6]. Methods based on selective regimes are strongly affected by trait dimensionality and independence [27] and are unsuited to investigate the evolutionary ‘history’ of convergence [10]. Metrics that necessarily require pre-selected states are strongly influenced by cases of uncertain categorization and by the choice of states. A few methods address convergence by assuming that a certain biological mechanism underpins the pattern [3]. Such methods cannot explain convergence that is not produced by directional processes, and are therefore inadvisable [22].

Here, we present a new method (available as the function *search*.*conv* in the R package RRphylo) which assesses convergence by testing whether phenotypes in distant clades in a phylogenetic tree are more similar to each other than expected by chance. The method works by computing the angle between the phenotypic vectors of the species as a measure of their similarity and allows identification of the clades (rather than just the species) that converge. We show through simulations that *search*.*conv* is remarkably powerful and fast. It does not require the convergent clades to be phenotypically unusual as compared to the rest of the tree. In addition, it has low (ca. 5%) Type I error rates (false positives).

We apply *search*.*conv* to three well-supported cases of morphological convergence, namely the independent adaptation to grazing in perissodactyl and artiodactyl mandibles, the evolution of the sabertooth morphology in machairodont cats and barbourofelids, and the evolution of distinct ecomorphs by Caribbean *Anolis*. The *seach*.*conv* function together with example files is available at https://github.com/pasraia/RRphylo.

## Materials and methods

The method is based on phylogenetic ridge regression, *RRphylo* [28]. With *RRphylo*, the phenotypic change between a node and a daughter tip along a phyletic line is described by the sum of individual contributions at each consecutive branch according to the equation $\Delta \text{y}={\vec{\beta }}_{1}l_{1}+{\vec{\beta }}_{2}l_{2}+\ldots .{\vec{\beta }}_{n}l_{n}$ where *n* equals the number of branches intervening between the node and the tip, ${\vec{\beta }}_{1}{}_{\ldots .n}$ are the vectors of regression coefficients (the evolutionary rates) at each branch, and *l*_*1…n*_ are the branch lengths. Regression coefficients are computed simultaneously for all the branches in the tree and independently for each variable (in the case of multivariate data), by applying to each of them a normalization factor λ which avoids fitting extreme *β* values and prevents multicollinearity [29].

Dealing with multivariate data, each species at the tree tips is represented by a phenotypic vector, including one entry value for each variable. Naming **A** and **B** the phenotypic vectors of a given pair of species in the tree, the angle θ between them is computed as the inverse cosine of the ratio between the dot product of **A** and **B**, and the product of vectors sizes:

$$\theta =arccos\frac{A•B}{\left|A||B\right|}$$

The cosine of angle θ actually represents the correlation coefficient between the two vectors [30]. As such, it exemplifies a measure of phenotypic resemblance [26]. Possible θ values span from 0 to 180 degrees. Small angles (i.e. close to 0°) imply similar phenotypes. At around 90° the phenotypes are dissimilar, whereas towards 180° the two phenotypic vectors point in opposing directions (i.e. the two phenotypes have contrasting values for each variable). For a phenotype with *n* variables, the two vectors intersect at a vector of *n* zeros (the origin of the axes in the 3D plot produced by using the S1 File). However, it is important to note that with geometric morphometric data (PC scores) the origin coincides with the consensus shape (where all PC scores are 0), so that, for instance, a large θ indicates the two species diverge from the consensus in opposite directions and the phenotypic vectors can be visualized in the PC space (S1 File).

**Fig 1:**
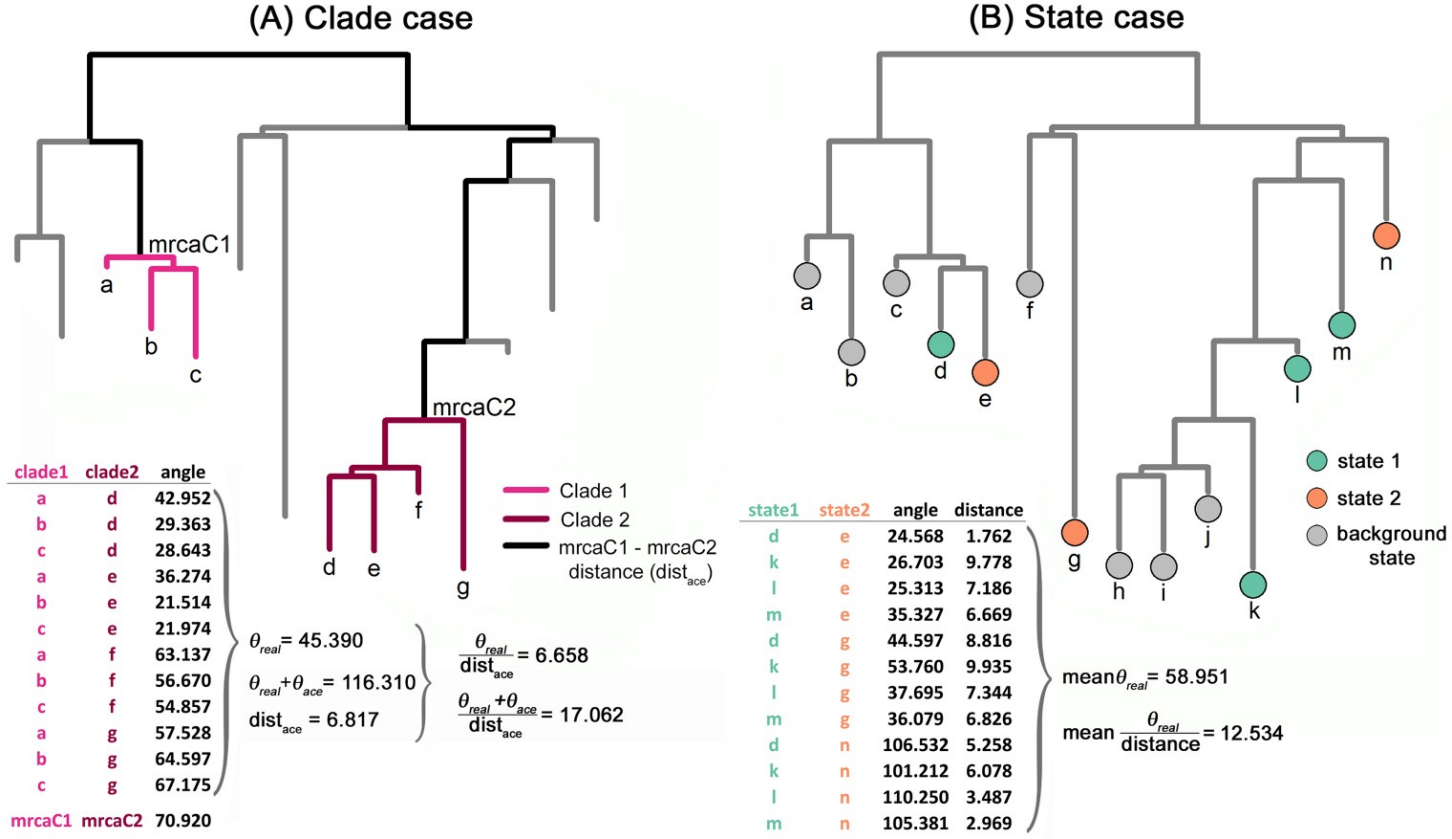
Hypothetical example illustrating how the *search*.*conv* function algorithm works. In the clade case (A), given any two monophyletic clades in the tree, the mean angle averaged over all possible combinations of two species (one per clade) is computed. This θ_real_ angle is divided by the distance between the most recent common ancestors to the respective clades, mrca1 and mrca2. Significance is assessed by comparing the result of this procedure to 1,000 randomly generated angles θ_random_ computed between species extracted by chance from the tree, divided by their respective distances. Angles are further computed between phenotypes at the mrcas. These θ_ace_ angles are summed to the corresponding θ_real_ to test whether convergence was already present at the beginning of clade history. Ancestral phenotypes are either estimated by *RRphylo* or provided by the user according to the fossil record. In the state case (B), θ_real_ are computed as in the clade case, but taking the mean angle between each combination of pairs of species (taken one per state), divided by their distance.

Under the Brownian Motion (BM) model of evolution, the phenotypic dissimilarity between any two species in the tree (hence the θ angle between them) is expected to be proportional to the age of their most recent common ancestor. Under convergence, this expectation is violated and the angle between species should be shallower than expected by their phylogenetic distance (see S1 File, selecting either ‘convergence’ or ‘convergence from similar ancestors’). We developed a new R function, *search*.*conv*, specifically meant to calculate θ values and to test whether actual θs between groups of species are smaller than expected by their phylogenetic distance. The function tests for convergence in either entire clades or species grouped under different evolutionary ‘states’ (Fig 1).

**Fig 2:**
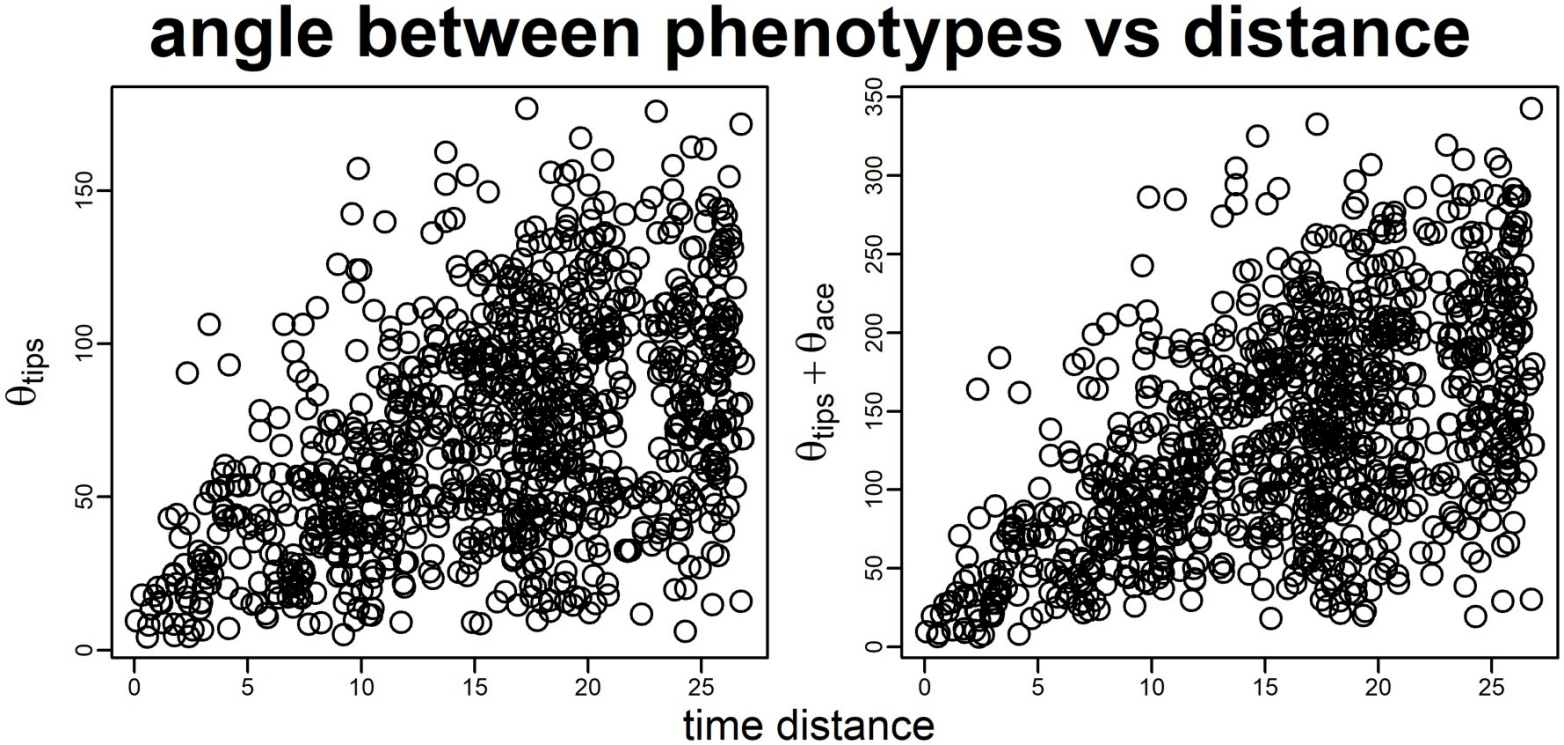
Plot of angles between phenotypic vectors versus time distance. The increase in the mean angle between the phenotypic vectors of all species pairs in the tree θ_tips_ and the distance between the species (left). The sum of θ_tips_ plus the angle between the phenotypes estimated at the first node above each tip θ_ace_ plotted against the distance between these nodes (right). The phenotype was generated according to the Brownian Motion model of evolution with sigma2 (the Browian rate) = 1. The tree is 100 species wide.

Given two monophyletic clades (subtrees) **C1** and **C2**, *search*.*conv* computes the mean angle θ_real_ over all possible combinations of pairs of species taking one species per clade. This θ_real_ is divided by the patristic (i.e. the sum of branch lengths) distance between the most recent common ancestors (mrcas) to **C1** and **C2**, **mrcaC1** and **mrcaC2**, respectively (Fig 1), to account for the fact that the mean angle (hence the phenotypic distance) is expected to increase, on average, with phylogenetic distance (Fig 2). To assess significance, *search*.*conv* randomly takes a pair of tips from the tree (*t1* and *t2*), computes the angle θ_random_ between their phenotypes and divides θ_random_ by the distance between *t1* and *t2* respective immediate ancestors (i.e. the distance between the first node *N1* above *t1*, and the first node *N2* above *t2*). This procedure is repeated 1,000 times generating θ_random_ per unit time values, directly from the tree and data. The θ_random_ per unit time distribution is used to test whether θ_real_ divided by the distance between **mrcaC1** and **mrcaC2** is statistically significant, meaning it is smaller than 5% of θ_random_ values the two clades are said to converge.

When testing convergence, researchers typically have species phenotypes and, ideally, a phylogenetic tree representing their relationships. This means that while it is usually possible to test convergence among species, it is generally not possible to identify entire clades evolving under convergence. In the real world, these clades actually coincide with **mrcaC1** and **mrcaC2** and their descendants. In *seach*.*conv*, we devised a strategy to identify **mrcaC1** and **mrcaC2**. In practice, given a pair of candidate nodes tested for the initiation of convergence, the phenotypes at **mrcaC1** and **mrcaC2** are estimated by *RRphylo*, and the angle between the ancestral states (θ_ace_) is calculated (see the angle between mrcas produced by using the S1 File). Then, θ_ace_ is added to θ_real_ and the resulting sum divided by the distance between **mrcaC1** and **mrcaC2**. The sum θ_ace_ + θ_real_ should be small for clades evolving from similar ancestors towards similar daughter phenotypes (see the average angle between tips, ‘mean.dir’, produced by using the S1 File). Importantly, a small θ_ace_ means similar phenotypes at the mrcas of the two clades, whereas a small θ_real_ implies similar phenotypes between their descendants. It does not mean, though, that the mrcas have to be similar to their own descendants. Two clades might, in principle, start with certain phenotypes and both evolve towards a similar phenotype which is different from the initial shape. This means that the two clades literally evolve along parallel trajectories (S1 File, select the option ‘convergence from similar ancestors’). Under *search*.*conv*, simple convergence is distinguished by such instances of convergence with parallel evolution. The former is tested by looking at the significance of θ_real_. The latter is assessed by testing whether the quantity θ_ace_ + θ_real_ is small (at alpha = 0.05) compared to the distribution of the same quantity generated by summing the θ_random_ calculated for each randomly selected pair of species *t1* and *t2* plus the angle between the phenotypic estimates at their respective ancestors *N1* and *N2* divided by their distance.

As with many other methods concerned with testing convergence (e.g. [10,18,31]), the *seach*.*conv* function suffers from the problem that ancestral states estimation entirely depends on the phylogenetic tree and data at hand and the evolutionary model used to fit the states. To help addressing this issue, under *search*.*conv* phenotypes at the nodes can be indicated directly by the user, when there is a specific hypothesis (i.e. real fossil specimens) about the phenotype of the most recent common ancestor to the clades. This is useful since the inclusion of fossil information increases power and reliability of comparative analyses of trait evolution [32,33].

Under *search*.*conv*, instances of convergence may be either assessed under the ‘automatic mode’ or specifying candidate node pairs. By default, *search*.*conv* runs the former, testing all clade pairs which are at least as distant as a one tenth of the tree size, counted as the number of nodes between their most recent common ancestors (i.e. clades 10 nodes apart for a 100 species tree). Alternatively, a time, rather than number of nodes, distance could be specified (we illustrate this procedure in the supplementary information and demonstrate via simulations how robust this alternative is). Although any minimum distance can be specified, it must be reminded that by testing too many node pairs at once potentially introduces Type I error inflation. We empirically found that this just becomes a problem by testing very small clades in very large trees. With the default option (i.e. nodes that are at least as distant as a one tenth of the tree size) Type I error inflation is negligible. As detailed below, we assessed the effect of phylogenetic distance on *search*.*conv* Type I and Type II error rates. Our expectation is that the closer the clades are on the tree, the harder it becomes to find convergence, as phenotypic similarity is best explained in this case by phylogenetic proximity.

Several candidate node pairs could map on the same region of the tree, because phenotypic values in close nodes are strongly autocorrelated (for instance, a candidate node pair could be represented by nodes n1 and n2, and another by the pair of nodes immediately bracketing n1 and n2). For each candidate node pair representing a statistically significant signal for convergence, *search*.*conv* performs the analysis of multivariate homogeneity of group dispersions by using the function *betadisper* in the R package vegan [34], calculates the average distance from group centroids for individual species in the clades, and orders candidate and significant node pairs (if they are > 1 in number) from the least variable to the most. The rationale is that under convergent evolution, species phenotypes are expected to deviate the least from group centroids, at least when the convergent states represent evolutionary attractors [1,2].

The clade-wise approach we have described so far ignores instances of phenotypic convergence that occur at the level of species rather than clades. The *search*.*conv* function is also designed to deal with this case. To do that, the user must specify distinctive ‘states’ for the species presumed to converge. The function will test convergence between any pair of given states. The species ascribed to a given state may belong anywhere on the tree or be grouped in two separate regions of it, in which case two states are indicated, one for each region. The former design facilitates testing questions such as whether all hypsodont ungulates converge on similar shapes, while latter aids in testing questions such as whether hypsodont artiodactyls converge on hypsodont perissodactyls.

If provided with such ‘states’ *search*.*conv* will calculate the mean θ_real_ between all possible species pairs evolving under a given state (or between the species in the two states presumed to converge on each other). The θ_random_ angles are calculated by shuffling the states 1,000 times across the tree tips. Both θ_real_ and individual θ_random_ are divided by the distance between the respective tips.

### Testing *search*.*conv* on convergence generated by unknown evolutionary processes

We assessed the power of *search*.*conv* using both simulation experiments and real cases. The first set of simulations reproduces the existence of phenotypically similar clades or species in distant regions of the tree. This corresponds to the traditional observation of entire clades converging towards similar ecomorphologies (e.g. adaptation to durophagy in the mandible and skull of borophagine canids and modern hyaenids, body shape in ichthyosaurs and dolphins).

We started by generating a paleontological (i.e. non ultrametric) tree with at least 80 species, by using the function *sim*.*bdtree* in the R package geiger (we set birth and death rates at 0.5 and 0.2, respectively [35]). Then, we produced a set of phenotypic data for the tree composed of three uncorrelated variables generated according to the BM model of evolution with variance (the Brownian rate) = 1, using the function *fastBM* in the R package phytools [36].

#### Clade case

**Fig 3:**
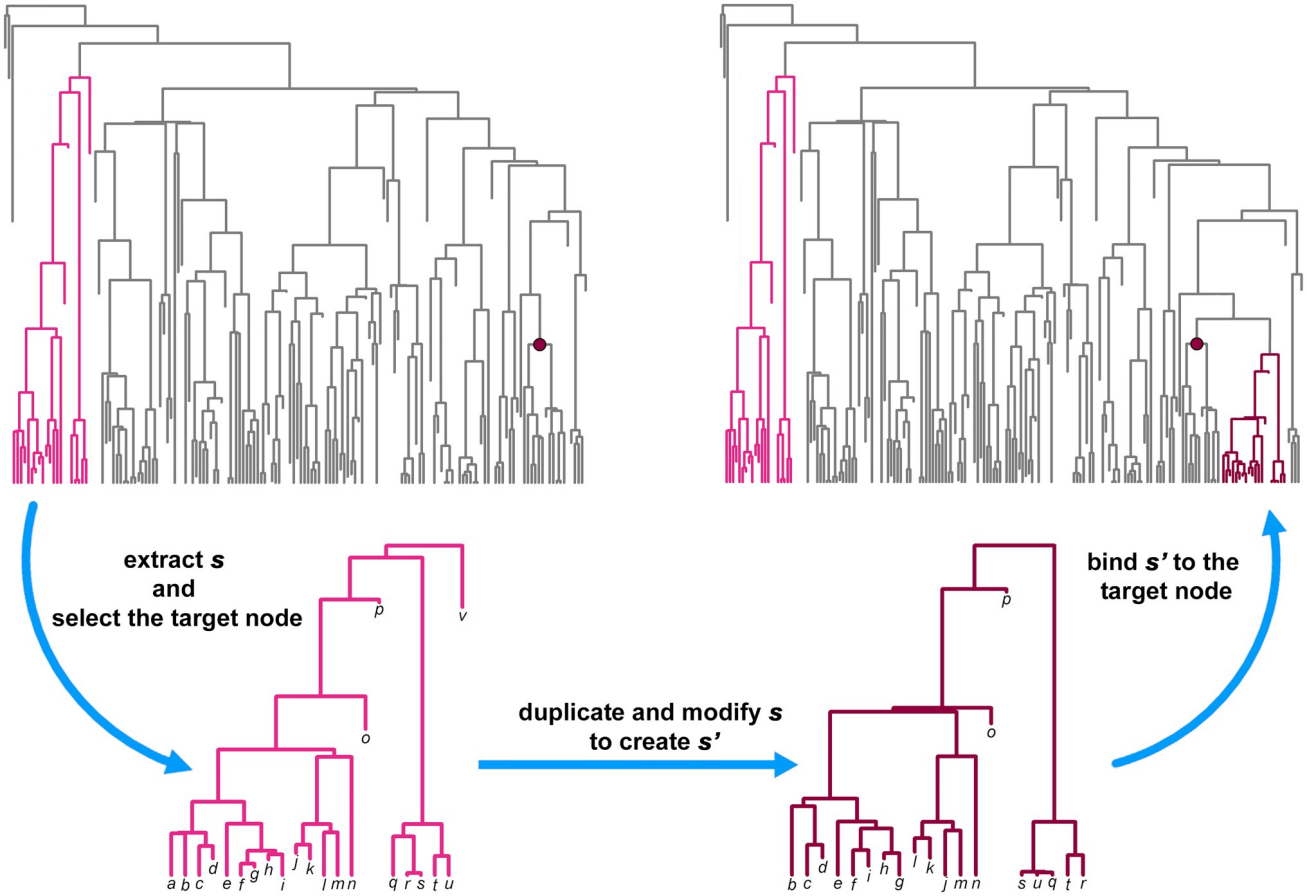
The procedure used to simulate convergence. Clades set to converge are colored. The focal clade (***s***) is indicated in bright pink, the modified clade (***s’*** dark pink) is grafted at the target node indicated by the dark pink dot.

To test for convergence between entire clades, our strategy was to select, duplicate, modify, and eventually attach a given clade and its phenotypes to the tree. First, we randomly selected a given subtree ***s***. Then, we changed its topology and branch lengths as to produce a new subtree ***s’***. The phenotypes in ***s’*** are similar but not the same as in ***s***. Eventually, ***s’*** and its phenotypes are grafted to a target node on the tree being at least as distant from ***s*** as one tenth of the tree nodes (Fig 3). Since the two subtrees have similar phenotypes in spite of being phylogenetically distant, they should be found to converge on each other.

To accomplish this procedure, we started by selecting ***s*** from within the tree among clades having as many as one tenth to one quarter of the tree tips. We deliberately avoided considering subtrees that are too young (i.e. more than 80% of the tree height in terms of distance from the root) given they would represent an unrealistic case of clades which have had very little time to evolve any convergence (Fig 3).

After modifying ***s*** to produce ***s’***, we assigned to their species phenotypes which are similar to each other and different from the rest of the tree, in order to avoid the new tree phenotype representing BM (which predicts no convergence). To produce the new phenotypes for ***s*** and ***s’***, we took the maximum value of each original variable (thereby creating a vector of maxima $\vec{m}$) and multiplied $\vec{m}$ by a random factor *f* ranging from 0.5 to 2 to generate a new vector $\vec{m^{\prime }}$. Then, for each subclade (***s*** and ***s’***) we produced a number *n* of phenotypes as long as the number of species of each subclade, using the function *jitter* in R. The variables in the new ***s*** and ***s’*** phenotypes were thus designed to have means equal to $\vec{m^{\prime }}$ and standard deviations equal to the standard deviations of the original variables.

**Fig 4:**
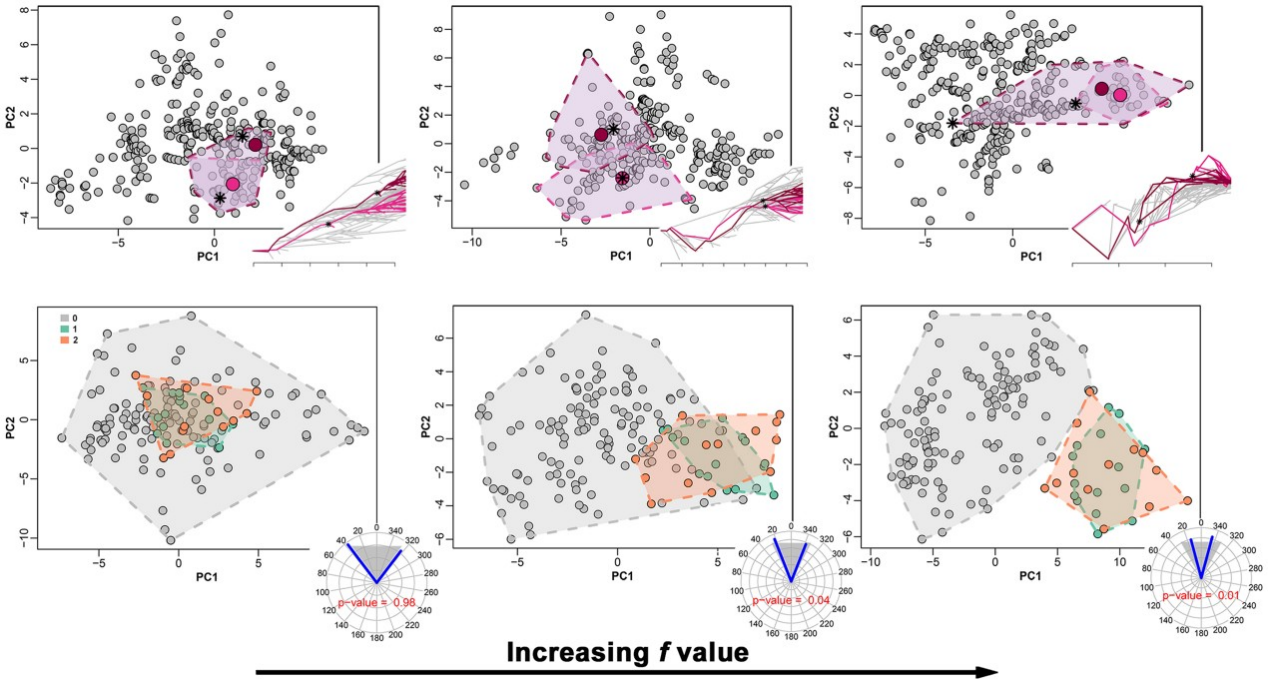
The effect of phenotypic similarity between clades set to converge and the rest of the tree phenotypes on *seach*.*conv* power. In each panel the PC1/PC2 plot of the tree phenotypes are reported. Clades set to converge are indicated by colored convex hulls. Upper row, clade case. Ancestral phenotypes (mrca*s*) of the clades set to converge are indicated by an asterisk. Large colored dots represent the mean phenotypes (group centroids) of the clades set to converge. A modified traitgram plot is added to the lower right corner in each figure, with branches belonging to the clades set to converge highlighted in color. Lower row, species belonging to states set to converge are indicated by colored convex hulls (*0* = background states, *1* and *2* are the states set to converge). To the lower right corner of the quadrants the circular plot reports the mean angle between states (blue lines) and the range of random angles (gray shaded area). The p-value for the convergence test is printed within the circular plots. The *f* values used to simulate the convergent clades are (from the left to the right): 0.2, 0.4 and 0.8.

With *f* > 1 the new phenotypes lay outside the range of the original, BM phenotypes, and the converse with *f* < 1. Thereby, we checked how ‘extreme’ the phenotypic values in ***s*** and ***s’*** have to be for *search*.*conv* to detect convergence (see Fig 4). Before attaching ***s’*** to the target node, we also dropped two species at random from the subtree and changed its topology and branch lengths by applying the function *swapONE* in RRphylo. By default, this function changes the topology for half of the tree tips and the length of half of the branch lengths (Fig 3). Eventually, the new subtree was rescaled on the height of the clade subtended by the target node (i.e. the maximum distance of its tips from the tree root equals the same distance for tips descending from the new node) so that both ***s*** and ***s***’ will terminate at the same distance from the root but will have very different heights (Fig 3).

In sum, the two clades set to converge have different topologies, branch lengths, ages and number of tips, only superficial phenotypic resemblance to each other, and may actually be very similar (phenotypically) to other clades in the tree (with *f* < 1, see Figs 3 and 4). While the distribution of phenotypes of the new tree departs from the BM expectation (which would violate the basic premise for convergent evolution) we deliberately produced phenotypes which are not too different from the rest of the tree phenotypes, to avoid testing *search*.*conv* with unrealistic or too obvious cases of convergence (see ref. [22] for a similar approach).

We performed *search*.*conv* on the tree and the attached phenotypic variables both by testing for convergence between all possible combinations of nodes (having proper size and distance) in the tree (the automatic mode) or by indicating target nodes (the specified clades mode). The entire procedure was repeated reducing the distance between convergent clades at three nodes only. In this latter case, the power of *search*.*conv* is expected to decrease because the phenotypic similarity between clades so close to each other is best explained by phylogenetic proximity rather than by phenotypic convergence. To assess the Type I error rates (i.e. the incidence of false positive instances of convergence found by *search*.*conv* when in fact there is none) we repeated the same procedure described above to modify the tree topology and branch lengths, and generated on this modified tree a BM phenotype. This way, no convergence is expected to occur between ***s*** and ***s’*** or anywhere else in the tree. The complete set of analyses was reiterated 100 times (i.e. for 100 different trees and phenotypes once to assess Type II and then again to assess Type I error rates).

We repeated the analyses to test the performance of *search*.*conv* with phenotypic variables generated by a non-BM process. To this aim, we rescaled the original tree in accordance with four different evolutionary models (“kappa”, “delta”, “lambda”, and “trend”) by using the function *rescale*.*phylo* in the package geiger [35]. The rescaled trees were used to produce multivariate phenotypes (formed by three variables each) generated according to these evolutionary models and then attached to the original (unscaled) tree. A fifth multivariate trait was generated according to the “drift” model (i.e. having a trend in the phenotypic mean over time) by using the function *setBM* in RRphylo. The procedure was repeated 25 times for each model by sampling model parameters (kappa ranging between 0 and 1, delta ranging between 0.1 and 3, lambda ranging between 0.1 and 1, trend ranging between -0.01 and 0.01, and drift with ds ranging between 0 and 1) at each repetition.

We checked whether the target subtrees are too similar to each other as compared to any other pair of clades in the tree, which would make the test look unreliably powerful and the simulation conditions naive. To this aim, we ran *RRphylo* on the modified tree and phenotype in order to estimate the ancestral phenotypes. Then, we calculated the multivariate Euclidean distance between all the ancestral phenotypes in the tree, to check whether the morphological distance between the two target (i.e. converging) nodes (***s*** and ***s’***) fell within the 95% confidence interval of the internode phenotypic distances. The entire procedure was repeated 100 times. At each repetition, we searched for cases of statistically significant convergence between all the nodes in the tree at least as distant from each other as the target nodes.

#### State case

To test for convergence among groups of species evolving under a single state, we randomly sampled a subgroup including up to one tenth of the number of species in the tree and set it to evolve under a given state. Species in this subgroup were then given new phenotypic values by applying the same procedure as described in the ‘clade’ case.

Similarly, to test for convergence between states, we repeated the procedure for two subgroups, set to converge morphologically on each other (Fig 1). Species in these subgroups were given new phenotypic values as we described in the ‘clade’ case. Yet, one of the two groups’ phenotypes were given twice the standard deviation as the original phenotype. The phenotypes thus fell into three different states: “background state” is the background state produced under BM, “state 1” and “state 2” are the states set to converge (Fig 1). The entire procedure was repeated 100 times.

### Testing *search*.*conv* on convergence generated by known evolutionary processes

The simulation sets described so far assume a pattern-based recognition of convergence, assessing whether phenotypically similar yet phylogenetically distant clades or species do represent convergent evolution regardless of the process generating convergence (see Supplementary S4 File for the R code). Two additional simulation sets address the power of *search*.*conv* to identify convergence by using an explicit process. We used Stayton’s [22] simulation design to this goal. In keeping with this, we started by using the function *sim*.*bd*.*taxa* in TreeSim [37] fixing the number of species at 26 (we set birth and death rates at 0.5 and 0, respectively). Then, we generated ten different phenotypic vectors according to the BM model. The phenotypic variance of the ten variables follows a broken-stick distribution [22]. Two to ten phenotypes were selected at each repetition and attached to the tree. Three species distant no less than three nodes from one another were selected from the tree and tested for convergence. Since all variables were generated under BM, no issue of convergence should be found by *search*.*conv*. Hence, this simulation set provides an assessment of *search*.*conv* Type I error rate. A second simulation set was applied to assess Type II error rate, still replicating Stayton’s procedure [22]. At this time, after producing the original, BM phenotypes as described above, three different lineages within the tree were randomly selected to evolve towards a common phenotype according to the OU process, with alpha (the strength of selection) randomly varying between 1 and 50 and theta (the phenotypic attractor) being 1.25 times the maximum values of the original BM phenotype. Both procedures were ran 1,000 times and the number of false positive and false negative instances provided by *search*.*conv* were recorded. Within the supplementary information, we illustrate these same simulations performed by using a time distance (rather than number of nodes distance) criterion (File S3).

### Real cases

We tested three real cases for possible instances of morphological convergence. They represent well-supported instances of morphological convergence during the evolution of the mammalian mandible (cases 1–2) and the colonization of the Caribbean islands by the lizard genus *Anolis* (case 3).

The first case concerns felids. Felids fall in two major ecotypes. Pantherine and feline cats possess robust, conical upper canines. A second ecotype was present in two extinct clades within the cat family, i.e. machairodonts and barbourofelids. The latter is the sister group to true felids. Machairodonts include three tribes [38], one with short and not particularly flattened upper canines, the Metailurini, a second with long, flattened upper canines often possessing crenulated margins, the Homotheriini, and a last tribe with exceptionally long, extremely flat upper canines with smooth margins, the Smilodontini. Smilodontini are the sister clade to Metailurini. Both Homotheriini and Smilodontini are “true” sabertooths [39]. The true sabertooth cats and barbourofelids present highly derived mandibular morphologies, specialized to confer these cats their unique killing behavior, including reduced dentition, low coronoid and condyle processes and protruding incisors [20]. We tested whether mandibular shape in the extinct sabertooth cat clade Machairodontini converges on mandibular shape in Barbourofelidae (the sister clade to all felids which is usually referred to as ‘false’ sabertooth cats). We used geometric morphometric data and the tree published in Piras et al. [38]. The geometric morphometrics data included 83 species and 711 specimens, and we chose 10 landmarks and 23 semi-landmarks to record the mandibular shape (S3 File). We used the first 15 eigenvectors to represent 95% of the cumulative shape variance explained. We ran this experiment with the ‘automatic’ procedure of *search*.*conv* (i.e. without specifying which clades to be tested).

We further explored the potential effect of specifying ancestral states in finding morphological convergence by applying *search*.*conv*. To this aim, we repeated the analysis by setting the ancestral mandibular phenotype of barbourofelids and machairodonts to be equal to *Barbourofelis fricki* and *Smilodon fatalis*, respectively.

We compared *seach*.*conv* to an existing method sought to address morphological convergence embedded in the R package convevol [40]. To this aim, we performed both *search*.*conv* (under the ‘state’ condition) and *convratsig* [40] by collapsing barbourofelids and sabertoothed cats under a single state. The function *convratsig* returns four distance-based metrics of convergence and their relative statistical significance obtained by means of randomizations. The C_1_ metric is the ratio of phenotypic distance between two (presumably convergent) tips (D_tip_) to the maximum phenotypic distance (D_max_) between any pair of taxa in those lineages. When the tips converge, C_1_ gets close to 1. The C_2_ metric quantifies the magnitude of convergence. It is computed as the difference between D_max_ and D_tip_. The C_3_ and C_4_ metrics are computed by dividing C_2_ by the total amount of morphological evolution intervening between the tips (i.e. the sum of phenotypic change along the tree branches) and by the total amount of morphological evolution in the entire clade defined by the mrca of convergent tips, respectively. All metrics rely on the estimation of ancestral states at internal nodes (reconstructed according to BM) and none of them include information about the timing for convergent evolution to take place [40].

The second case study was based on hooved mammals (Ungulatomorpha). Hooved mammals fall into two major feeding categories, that is browsing on soft vegetable matter, and grazing on harder vegetable material, typically grasses, whose leaves are rich in silica and therefore result in increased wear rate of the molar tooth crowns. Browsing is typical of most Palaeocene and Eocene ‘ungulates’ and persists today in most deer, tragulids and other small-bodied forms [41]. With the emergence of grasslands and the spread of grasses, the inclusion of grasses in the diet became widespread in herbivorous mammals [42,43] and is responsible for the rapid diversification of grazing artiodactyls [16]. In morphology, the dietary shift from soft (browsing) to hard and fibrous (grazing) plant material is accompanied by profound changes in the skull and mandible, including the acquisition of high-crowned (hypsodont) molars, longer snout, and deeper mandible [44–46]. This pattern is present in equids, and also appeared several times among Pecora. Nonetheless, true grazing is restricted to a minority of species, most of them being properly defined as mixed-feeders consuming both grasses and soft material [44].

The data were obtained from 353 images in lateral view taken from the scientific literature or directly from specimens (see S2 File for full details), representing 205 species. On each image we recorded nine landmarks to register mandibular shape and analyzed shapes by means of geometric morphometrics (see S3 File for details). We used the five largest eigenvectors, as they represent 95% of the cumulative shape variance explained. The ungulate tree was assembled from literature [16,46,47]. We considered individual species as either grazing artiodactyls, grazing perissodactyls, or “others” (i.e. non convergent) depending on their molar morphology (i.e. degree of hypsodonty) and tested whether grazing ungulates from different parts of the tree converged on similar mandibular morphologies by using the ‘state’ approach.

The third real case pertains to extant lizards of the genus *Anolis*. The genus includes more than 400 species distributed in the Neotropical region and the Caribbean. Insular anoles fall into six distinct ecomorphs which have been intensely studied as a classic example of convergent evolution [19]. The data include a 100 species wide tree for *Anolis* lizards living on the main islands of the Greater Antilles, and 11 phylogenetic principal components extracted analyzing lizards body shapes [48,49] (see Supplementary S4 File for the R code). Six species do not fall into any ecomorph category and are therefore not expected to converge.

## Results

### Testing convergence generated by unknown evolutionary processes under the automatic mode

The average tree size in the simulation experiments was 192.14 tips (range 156–247). The clades set to converge varied from 17 to 44 species (average 26.74). On average, the heights of the clades set to converge were 64.39% the tree height (range 10.33%-87.76%). The Euclidean distance between ***s*** and ***s’*** respective mrcas phenotypes falls with the 95% confidence intervals of the distribution of inter-node distances in the tree 96% of the times. The distance between the convergent clades was, on average, 98% of the tree height (range 30.14%-166.23%). Despite this great variation in convergent clade size, distance and height, under the automatic mode the Type II error (the rate of false negatives) is as low as 6%. Type I error (false positive) rate is similarly low at 4%. We analyzed the effect of tree size, *f*, and convergent clades’ relative size and distance (that is clade size and the distance between the **mrcaC1** and **mrcaC2** divided to the tree size and height, respectively) on the likelihood to find convergent clades, by regressing these metrics against the p-value calculated for θ_real_ over 100 simulations. The effect of relative clade distance is negative and almost significant (p = 0.063) whereas *f* is positive and significant (p = 0.037), meaning that the likelihood of finding convergence increases for clades with distinctive phenotypes and relatively distant from each other on the tree as expected (Fig 4).

As expected, when the simulations were repeated with clades separated by only three nodes, Type I error is 0%, whereas Type II error increases to 54%. These results indicate that *search*.*conv* does not find convergence between clades that are very close to each other on the tree, whose phenotypic resemblance is best explained by phylogenetic proximity rather than convergence.

### Testing convergence generated by unknown evolutionary processes by specifying candidate clades

The power of *search*.*conv* to correctly identify the convergent clades when they are specified by the user (i.e. both θ_real_ and θ_real_ + θ_ace_ are significant) is 71%. However, considering cases when species phenotypes (θ_real_) are found to be significantly convergent but θ_real_ + θ_ace_ is not, the identified mrca*s* for the clades found to converge were correct 88% of the time, within 2 nodes distance from the convergent clades’ mrcas. *search*.*conv* often identifies nodes which are very close to the ‘real’ mrcas rather than the ‘real’ mrcas themselves. We found this usually depends on the balancing between the clade set to converge and its sister node, and the strong phenotypic autocorrelation between these clades (because a given clade necessarily includes all of the descendants of its daughter node). When the sister to the real mrca is made up of very few species *search*.*conv* usually identifies a younger node than the real mrca. Whichever exact mrca pair is identified, 97.5% of the species set to converge are, on average, found to do so.

The Type I and Type II error rates of *search*.*conv* (automatic mode) are little influenced by how the phenotypes are simulated. The Type I error (the percentage of false positives) remains remarkably low (Table 1). However, some types of phenotypes (most notably ‘drift’) present high Type II error rate (Table 1).

**Table 1:** Type I and Type II error rates.

	Type II error	Type I error
**Phenotype type**		
browian	6.00%	4.00%
kappa	4.00%	0.00%
delta	12.00%	0.00%
lambda	0.00%	0.00%
trend	12.00%	4.00%
drift	16.00%	4.00%

Type I and Type II error rates with phenotypes simulated according to different evolutionary models.

### Testing convergence generated by unknown evolutionary processes using evolutionary states

By using the ‘state’ specification, the Type I error rate is 5%, either within or between states. Type II error of *search*.*conv* is 1% when testing for convergence within a group and 6% testing two different states for convergence on each other. We did not find a significant regression between the rank of θ_*real*_ and *f* across 100 simulations (within state p = 0.104; between states p = 0.882). This is not surprising because under the state case species evolving under a single state appear randomly across the tree, hence the effect of *f* transformation is diffused rather than focusing on a single clade.

### Testing convergence generated by known evolutionary processes

We found 47 instances of convergence among groups of three randomly selected species out of 1,000 simulations with phenotypes designed to evolve under the BM model. This means that the Type I error rate of *search*.*conv*, under this condition, is 4.7%. By using the OU process to model convergence, we found *search*.*conv* fails to recognize convergence 45 times out of 1,000 simulation. The corresponding figure for Type II error rate (4.5%) is below the nominal alpha level (5%). By using time distances to select clades for convergence, we found Type I error rate as low as 2% and Type II rate at 4.9% (see supplementary information).

### Real case scenarios

#### Felid mandibles

**Fig 5:**
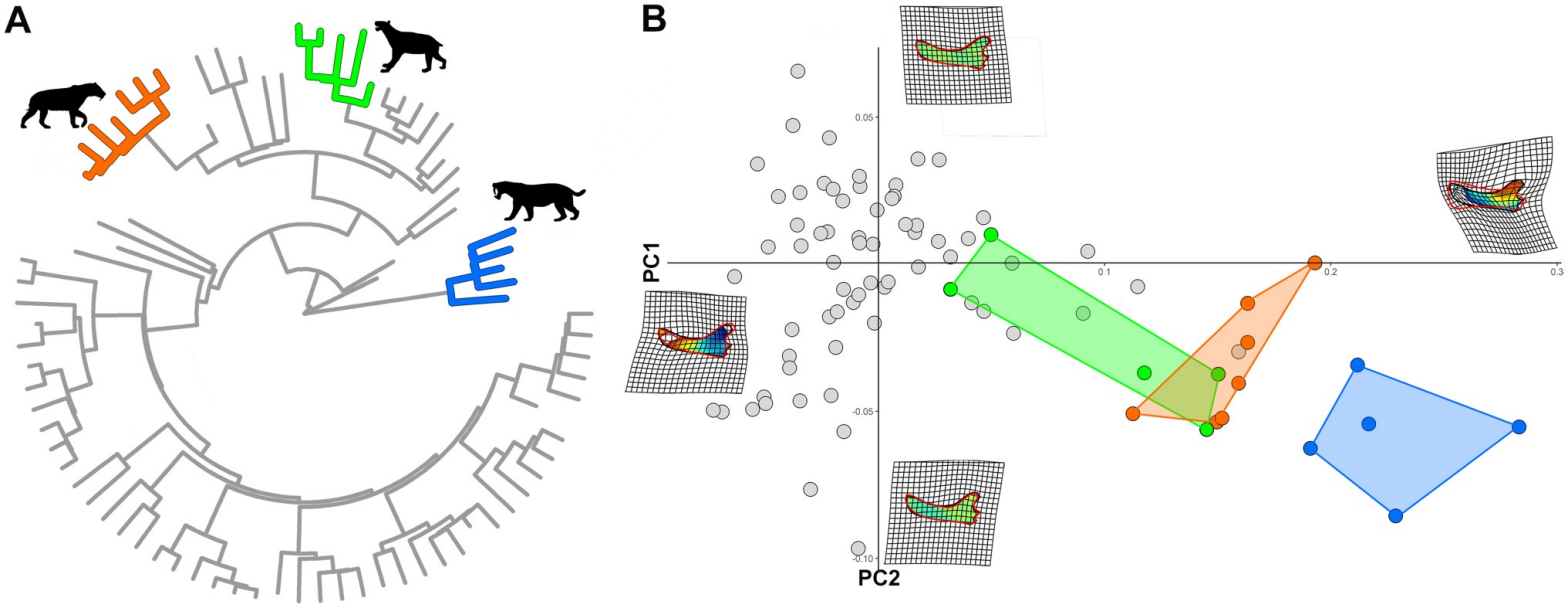
Convergence among mandibular shapes in felids. A) The clades found to converge were Homotheriini (orange) and Barbourofelidae (blue) and Smilodontini (green). B) PC1/PC2 plot showing the position of the convergent clades compared to the rest of the tree. Deformation grids are shown at the extremes of both axes. The silhouette for *Homotherium* was available for reuse under the Creative Commons Attribution 3.0 Unported (https://creativecommons.org/licenses/by-sa/3.0/) at http://phylopic.org/image/c6c2d17b-56b3-4c87-97c4-cd2b7de365fa/ (image by Zimices). The silhouettes for *Smilodon* and *Barbourofelis* are our own work.

When testing for convergence between clades, we found two instances of convergent morphological evolution, both pertaining the same clade, Barbourofelidae. The latter includes false saber-toothed cats of the genera *Barbourofelis* and *Albanosmilus*. They were found to be convergent on both Smilodontini and Homotheriini within machairodonts, which represent the true sabertoothed cats (Fig 5). It is noteworthy that *search*.*conv* effectively failed to find convergence between barbourofelids and Metailurini (Fig 5), which form a clade of machairodont cats sister to Smilodontini but did not possess the full sabertooth morphology. The mean angle between barbourofelids and Smilodontini is 29.93 degrees (Table 2A). The angle between their ancestors is 21.50 degrees. Both θ_*real*_ and θ_*real*_ + θ_*ace*_ are statistically smaller than expected by chance (p = 0.009 for both). This suggests that the two clades evolved along parallel trajectories. The angle between barbourofelids and Homotheriini is 43.09 degrees, the angle between their reconstructed ancestors is 39.09 degrees, and both θ_*real*_ and θ_*real*_ + θ_*ace*_ are statistically significant (p = 0.019 and 0.011, respectively; Table 2A). The computational time was 145 seconds.

**Table 2:** The results of *search*.*conv* applied to felid mandibular shape.

**A. *ACE estimated***							
candidate node pairs		θ_ace_	θ_real_	distance (# nodes)	distance (years * 10^6^)	p (θ_real_)	p (θ_real_+θ_ace_)
Smilodontini	Barbourofelidae	21.50	29.93	9	43.5	0.01	0.01
Homotheriini	Barbourofelidae	39.09	43.09	9	40.8	0.02	0.01
**B. *ACE indicated***							
candidate node pairs		θ_ace_	θ_real_	distance (# nodes)	distance (years * 10^6^)	p (θ_real_)	p (θ_real_+θ_ace_)
Smilodontini	Barbourofelidae	34.44	29.93	9	43.5	0.01	0.01
Homotheriini	Barbourofelidae	51.67	43.09	9	40.8	0.03	0.03

The results of *search*.*conv* applied to felid mandibular shape, either by estimating ancestral phenotypes by RRphylo (A), or specifying the ancestral phenotypes to all barbourofelids and all machairodonts to be equal to the phenotype of *Barbourofelis fricki* and *Smilodon fatalis* (B), respectively. ACE = ancestral character state (i.e. the ancestral phenotype), p (θ_real_) the significance of convergence test restricted to species only, p (θ_real_ + θ_ace_) the significance of convergence test for the θ_real_ + θ_ace_ sum.

By using the mandibular shapes of *Barbourofelis fricki* and *Smilodon fatalis* as the ancestral states to all barbourofelids and machairodonts, respectively, the results are similar to those obtained without specifying phenotypes at the mrca nodes (Table 2B, S3 File), and this may help explaining the good performance of *search*.*conv* in finding the correct position, hence the true identity, of converging clades.

By performing the analysis collapsing machairodonts and barbourofelids under a single state, *search*.*conv* produced a small and significant mean angle (19.93 degrees, p = 0.001) between convergent species. The computational time was 44 seconds. This latter analysis performed by using *convratsig* [40] produced significant results for all the measures (Table 3). The computational time was 21h 48’ 7”.

**Table 3:** The results of *convratsig* applied to felid mandibular shape.

	value	p-value
**C1**	0.259	0
**C2**	0.058	0
**C3**	0.110	0
**C4**	0.013	0

Distance-based measures of convergence and relative significance level as derived by the function *convratsig* in the R package convevol.

#### Grazing ungulate mandibles

We performed *search*.*conv* once taking grazers as a single group, then considering grazing artiodactyls and grazing perissodactyls separately.

**Fig 6:**
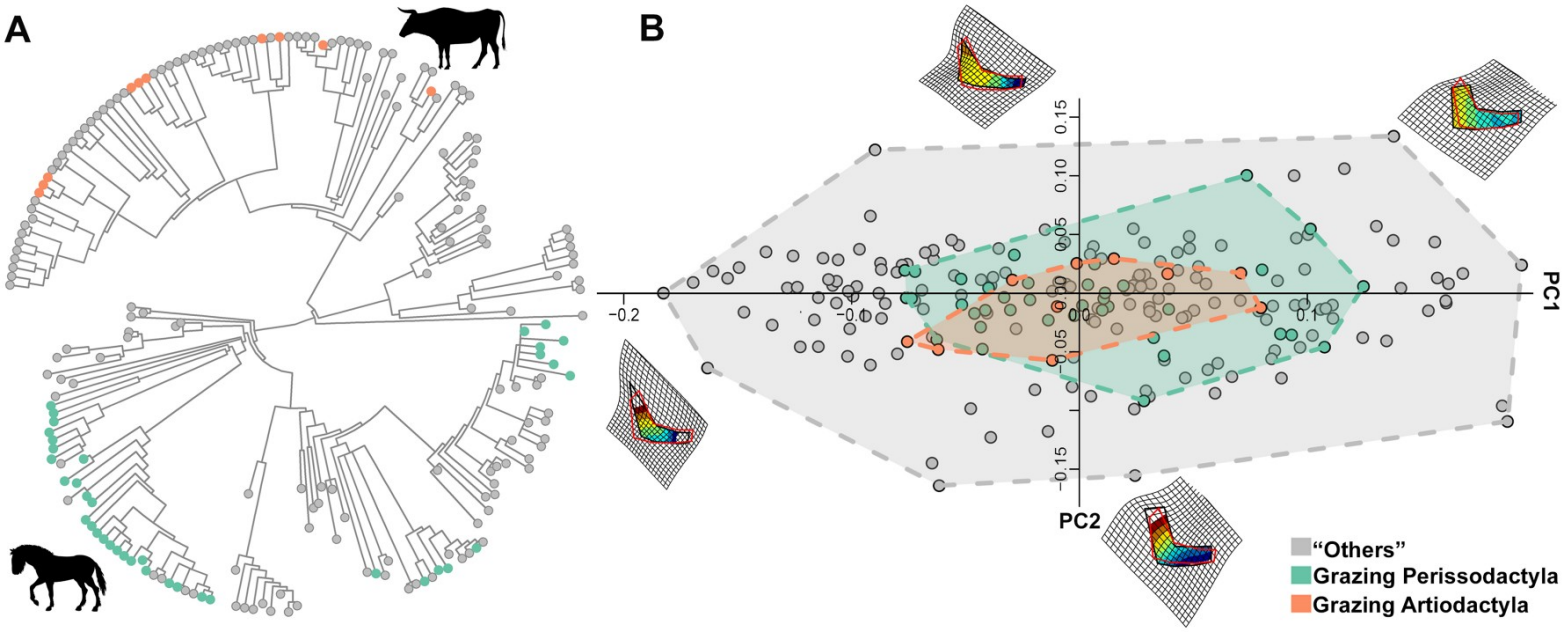
Convergence among mandibular shapes in ungulates. A) The distribution of individual species per state (gray = background state (others), orange = grazing artiodactyls, green = grazing perissodactyls) on the ‘ungulate’ tree. B) PC1/PC2 plot showing the position of the convergent states (grazers) compared to the rest of the tree. Deformation grids are shown at the extremes of both axes. Animal silhouettes were available under Public Domain license at phylopic (http://phylopic.org/). Specifically, *Bos primigenius* (http://phylopic.org/image/dc5c561e-e030-444d-ba22-3d427b60e58a/) image by DFoidl (modified by T. Michael Keesey) and *Equus ferus* (http://phylopic.org/image/85d95128-912c-427a-9542-138e1dbf5651/) image by Mercedes Yrayzoz (vectorized by T. Michael Keesey) are available for reuse under the Creative Commons Attribution 3.0 Unported (https://creativecommons.org/licenses/by-sa/3.0/).

The mean angle between all grazers collapsed under a single state is 69.62 degrees. This is significant at p = 0.041 (see S3 File for figure). The mean angle between grazing artiodactyls and grazing perissodactyls is 77.53 degrees. Although large, we found this angle is less than expected by chance (p = 0.001, Fig 6). In fact, the angle θ_real_ increases by 0.51 degrees per million year between grazing artiodactyls and grazing perissodactyls (which are separated by some 152 million years of independent evolution on the ‘ungulate’ tree, i.e. at least twice as much as the inferred age of the most recent common ancestor to all ‘Ungulatomorpha’). This same figure is 0.71 degrees per million year between grazing perissodactyls and “others” and 0.65 between grazing artiodactyls and “others”.

#### Caribbean *Anolis*

By using *search*.*conv*, we found significant convergence in 5 out of the 6 ecomorphs traditionally recognized for insular anoles (Table 4, see S3 File for figure).

**Table 4:** Results of convergence within *Anolis* ecomorphs. The left columns represent the results obtained by applying *search*.*conv*. The last two rightmost columns are the corresponding results pertaining to the metrics C1 and C5, retrieved from [22]. mean angle = the mean angle between species within the ecomorph; mean angle by time = the mean angle between species within the ecomorph divided by time distance; p mean angle = significance level for mean angle; p mean angle by time = significance level for mean angle by time; p-value C1 = significance level for the C1 measure [22]; p-value C5 = significance level for the C5 measure [22].

Ecomorph	*search*.*conv*				Stayton 2015	
	mean angle	mean angle by time	p mean angle	p mean angle by time	p-value C_1_	p-value C_5_
Trunk-ground	44.064	32.204	<0.001	<0.001	0.008	0.120
Grass-bush	35.855	24.835	<0.001	<0.001	<0.001	0.386
Crown-giant	20.814	36.305	<0.001	0.003	<0.001	<0.001
Trunk-crown	68.635	45.403	0.002	0.040	0.186	0.011
Twig	30.050	19.695	<0.001	<0.001	<0.001	0.445
Trunk	41.289	43.735	<0.001	0.119	0.252	0.002
None	87.996	53.344	0.317	0.991	0.255	0.763

We found convergence in 5 out of 6 different ecomorphs, the only exception being ‘trunk’ anoles. The *Anolis* species that cannot be ascribed to any ecomorphs are, unsurprisingly, not found to converge. By using the C1 metric, Stayton [22] found 4 of 6 ecomorphs converging. By using the metric C5, convergence is found in 3 ecomorphs. Species not ascribed to an ecomorph were not found to converge for either of the metrics.

## Discussion

Evolutionary convergence has been the focus of many evolutionary studies [6,21]. Morphological convergence arises from adaptation to similar niches by different lineages, which can be separated geographically, phylogenetically and temporally [12], although different processes such as phylogenetic and developmental constraint, and even chance may produce the same pattern [50–53].

There are several methods available in the literature to test the hypothesis of morphological convergence. Most of them rely on the basic assumption that convergence implies stronger phenotypic resemblance than expected by phylogenetic distance. Although the method we propose here, *search*.*conv*, makes this same assumption, it additionally helps identifying entire clades evolving towards similar shapes and recognizing whether they actually converge from different starting points (which we deem simple convergence) or evolved along parallel trajectories (note that under Adams and Collyer’s phenotypic trajectory analysis there is no expectation about how large the angle between a pair of phenotypic vectors should be [26]). Our procedure to identify convergence between clades is at least as dependent on ancestral state estimation as many other approaches (e.g. [6,10,23,54]). However, in *search*.*conv* it is possible to indicate specific phenotypes at nodes, if they are known from the fossil record, which can reduce the impact of ancestral states estimation.

We demonstrate *search*.*conv*, which is embedded in the R package RRphylo, is robust, has low Type I and Type II error rates, and is very fast even with reasonably large trees. Although the mrcas set to converge are not always found with precision under the automatic mode, the species actually set to converge are correctly identified up to 97.5% of the time, further demonstrating the selection of clade pairs is reasonably precise. When the starting phenotype was modelled to follow an evolutionary model other than BM, the function remains powerful, perhaps with the exception of the ‘drift’ (a trend in the mean phenotype over time) case. The lower performance of *seach*.*conv* on ‘drift-ed’ phenotypes might depend on the fact that ancestral state estimation is bounded by the actual phenotypes at the tips, making it evident how highly informative the specification of ancestral phenotypes could be.

We successfully applied *search*.*conv* to mandibular shape evolution in mammals in two different real cases and to Caribbean islands anole ecomorphs. The first real case study regards the evolution of mandibular shapes in felids. We found “true” sabertooths (Homotheriini and Smilodontini) independently converge on barbourofelids in their mandible morphology. Intriguingly, Metailurini (i.e. “false” sabertooths) which is nested within the machairodont family, were not found to converge on barbourofelids under the automatic mode. This means *search*.*conv* successfully excluded the false sabertooths from the convergence pattern despite their phylogenetic position close to other “true” sabertoothed machairodont cats [55,56].

We used the felid data to compare *search*.*conv* to convevol’s *convratsig* function. While both functions recognize the same pattern, *search*.*conv* was found to be three orders of magnitude faster, which could be crucial when it comes to assessing convergence with uncertain state categorization, or to taking the effect of phylogenetic uncertainty into account, as this implies repeating the analyses dozens of times by using different phylogenetic hypotheses.

The second real case application, performed with the ‘state’ approach, relates to the evolution of hypsodonty due to grass feeding in ‘ungulates’. Grazing adaptations in the mandible evolved independently in horses (genus *Equus*) and several bovid lineages, most notably among antelopes. We found evidence for convergent evolution between *Equus* and strictly grazing bovids, such as *Bison*, *Bos*, and *Alcelaphus*. This is especially noteworthy considering that the paleontological tree we used includes a number of non-grazing equids, such as hipparionoid horses and browsing anchitheriine equids, plus several extinct rhinos and tapirs which were all browsers. This demonstrates the method was able to find convergence among grazers despite the effect of phylogeny and body size on mandibular shape variation [46].

The final real case pertains to *Anolis* ecomorphs. We found evidence for convergence in all of them but the ‘trunk’ ecomorph species. Intriguingly, five of the six ‘trunk’ groups belong to a single monophyletic clade, indicating that the trunk ecomorph evolved only twice, once for a single clade only present on Hispaniola and then again when Cuban *Anolis loysiana* converged on them.

Compared to other statistical procedures used to test for morphological convergence, *search*.*conv* offers the possibility to test convergence between entire clades, and allows testing specific ‘states’ sparsely distributed across the tree. In addition, being much faster than alternative approaches, *seach*.*conv* allows exploring the potential effect of phylogenetic uncertainty and use of fossil phenotypes as ancestral states, that can be crucial in the presence of non-Brownian processes. It must however be noted that not all cases of “convergence” may be explored best with *search*.*conv*. There are several instances reported in literature of convergence between closely related clades and even single species with close phylogenetic proximity. We provide a test which is useful to find instances of large-scale morphological resemblance between distant clades that are generally referred at as either ‘convergent’ or just cases of iterative evolution. Caution must be applied to the choice of the ancestral phenotype in the presence of strong phenotypic drift.

## Supporting information

S1 File Angles theta for ancestors and tips of two clades evolving with or without convergence. The R code produces a 3D plot showing the distribution of phenotypes for a > 100 species tree. Two clades are selected to evolve convergent tip phenotypes (simple convergence), convergent tip phenotypes starting from ancestors which are similar to each other (convergence and parallel trajectories) or no convergence. At running the code, the user interactively chooses which kind of simulation to perform and how much the convergent clades have to be different (phenotypically) from the rest of the tree.
(R) Click here for additional data file.

S2 File Supplementary information for ‘ungulates’. State attribution with references and source for images used in GMM.
(XLSX)

S3 File Supplementary material. Details of the methods and additional results.
(DOCX)

S4 File Convergence simulations. R code to perform the simulations.
(R) Click here for additional data file.
