# Correction: A new, fast method to search for morphological convergence with shape data

**DOI:** 10.1371/journal.pone.0252264

**Published:** 2021-05-20

**Authors:** Silvia Castiglione, Carmela Serio, Davide Tamagnini, Marina Melchionna, Alessandro Mondanaro, Mirko Di Febbraro, Antonio Profico, Paolo Piras, Filippo Barattolo, Pasquale Raia

There are errors in the author affiliations. The correct affiliations are as follows:

Silvia Castiglione^1^, Carmela Serio^1^, Davide Tamagnini^2^, Marina Melchionna^1^, Alessandro Mondanaro^1,3^, Mirko Di Febbraro^4^, Antonio Profico^5^, Paolo Piras^6,7^, Filippo Barattolo^1^, Pasquale Raia^1^

1 Dipartimento di Scienze della Terra, dell’Ambiente e delle Risorse, University of Naples Federico II, Napoli, Italy 2 Dipartimento di Biologia e Biotecnologie “Charles Darwin”, Sapienza Università di Roma, Rome, Italy 3 Dipartimento di Scienze della Terra, University of Florence, Firenze, Italy 4 Dipartimento di Bioscienze e Territorio, University of Molise, Pesche, Isernia, Italy 5 Dipartimento di Biologia Ambientale, Sapienza Università di Roma, Rome, Italy 6 Dipartimento di Ingegneria Strutturale e Geotecnica, Sapienza Università di Roma, Rome, Italy 7 Dipartimento di Scienze Cardiovascolari, Respiratorie, Nefrologiche, Anestesiologiche e Geriatriche, Sapienza Università di Roma, Rome, Italy
