# Supplementary material for: A new, fast method to search for morphological convergence with shape data
Source: PLoS One. 2019 Dec 27;14(12):e0226949. doi: 10.1371/journal.pone.0226949 (PMC6934287; doi:10.1371/journal.pone.0226949)
Supplement: S3 File — Details of the methods and additional results. (DOCX) [file pone.0226949.s003.docx]

**A new method to search for morphological pattern-based convergence with shape data**

Silvia Castiglione^1^, Carmela Serio^1^, Davide Tamagnini^2^, Marina Melchionna^1^, Alessandro Mondanaro^1,3^, Mirko Di Febbraro^4^, Antonio Profico^2^, Paolo Piras ^5,6^, Filippo Barattolo^1^, Pasquale Raia^1^*

**Data and trees**

We collected 353 images of “*Ungulatomorpha*” mandibles representative of 205 species of Artiodactyla and Perissodactyla. We assembled a phylogenetic tree from several sources (Raia et al 2010, Raia et al. 2013). For felids, we used shape data and phylogeny published in Piras et al 2018.

**Geometric Morphometric - GMM**

Mandible shape data were elaborated by means of Geometric Morphometrics. Such technique allows to extract shape information from anatomical objects (the hemimandible in this case) starting from the spatial coordinates of a set of homologous landmarks. Generalized Procrustes Analysis retrieves landmarks coordinates and eliminates non-shape variation by means of rotation, scaling, and translation procedures. Relative Warps Analysis is applied to these new aligned coordinates to decompose shape variation into principal axes (shape eigenvectors) called Relative Warps (RWs) under the GMM jargon.

*
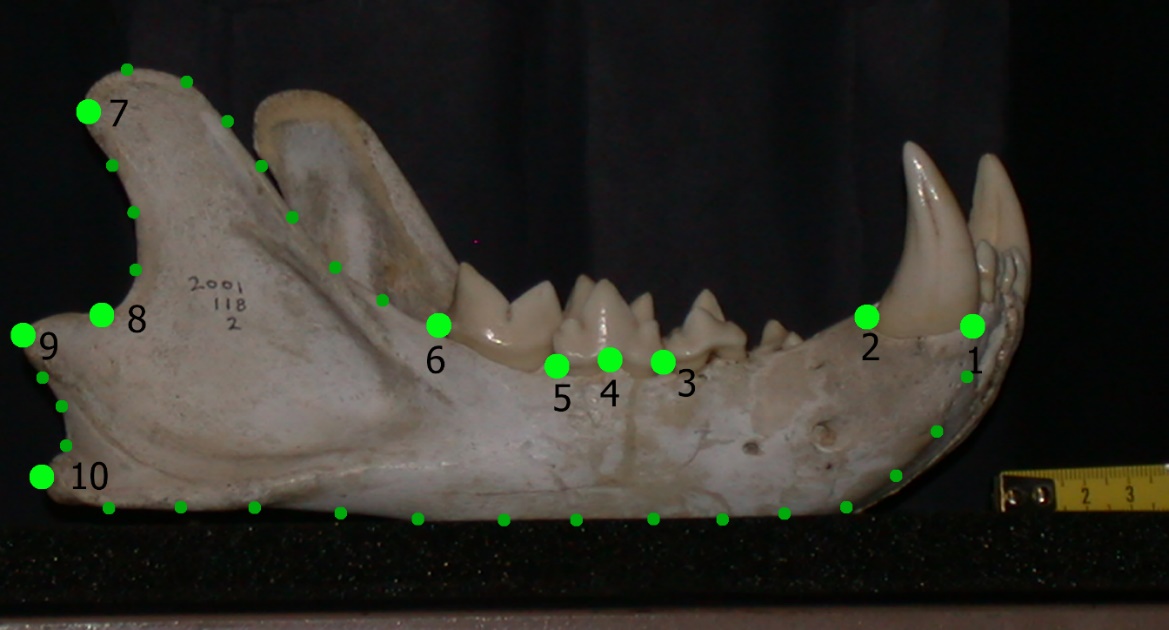
****GMM Carnivores***

**Supplementary Figure S1- Large green dots represent landmarks applied to a *Panthera leo* hemimandible. Landmark definitions: 1, 2) anterior and posterior tips of canine alveolus, respectively; 3, 4, 5) anterior, median, posterior tips of p4 alveolus; 6) posterior tip of m1 alveolus; 7) lowest point of coronoid process; 8, 9) limits of articular process; 10) posterior tip of angular process.**

In the case of felids, GMM produces 74 RWs. In particular, RW1-15 explain 95.05% of cumulative variance. We used them for the convergence test subsequent analysis.

|  | **eigenvalues** | **%Variance** | **%Cumulative** |
| --- | --- | --- | --- |
| **RW1** | **2.80E+03** | **44.70%** | **44.70%** |
| **RW2** | **1.55E+03** | **17.33%** | **62.03%** |
| **RW3** | **1.33E+03** | **10.55%** | **72.58%** |
| **RW4** | **1.20E+03** | **5.12%** | **77.69%** |
| **RW5** | **1.03E+03** | **4.21%** | **81.91%** |
| **RW6** | **9.64E+02** | **2.93%** | **84.84%** |
| **RW7** | **9.11E+02** | **2.32%** | **87.16%** |
| **RW8** | **8.85E+02** | **1.50%** | **88.66%** |
| **RW9** | **8.48E+02** | **1.41%** | **90.07%** |
| **RW10** | **8.09E+02** | **1.10%** | **91.18%** |
| **RW11** | **7.19E+02** | **1.01%** | **92.19%** |
| **RW12** | **6.48E+02** | **0.84%** | **93.02%** |
| **RW13** | **6.00E+02** | **0.75%** | **93.77%** |
| **RW14** | **5.07E+02** | **0.67%** | **94.44%** |
| **RW15** | **4.68E+02** | **0.57%** | **95.01%** |

Supplementary Table S1- RWA results for Felids. Eigenvalues: eigenvalues of the Covariance matrix; %Variance: variance for each RW; %Cumulative: cumulative variance.


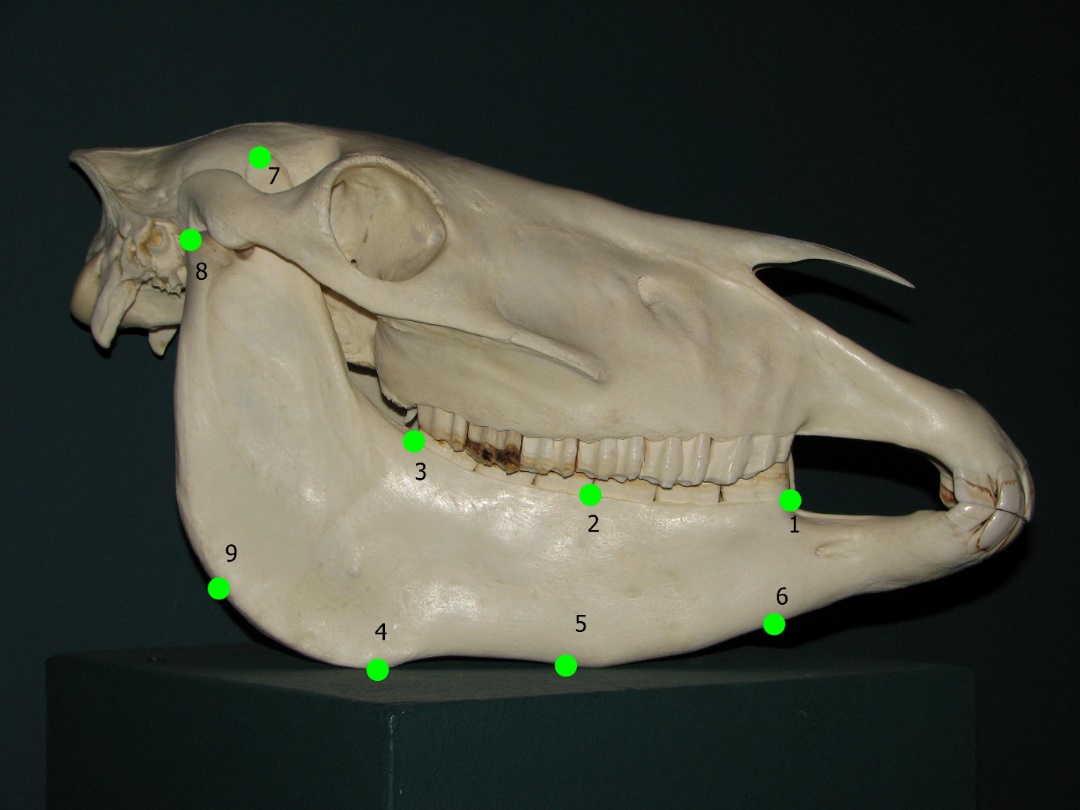
***GMM Ungulates***

**Supplementary Figure S2- Landmarks configuration for “*Ungulatomorpha*”, here applied on a mandible of *Equus ferus*. Landmark definitions: 1, 2) premolar tooth row; 2, 3) molar tooth row; 4, 5, 6) orthogonal projection of landmark 1, 2 and 3; 7) the highest point of coronoid process; 8) highest point of condyle; 9) 45° projection of landmark 3,4.**

In the case of ‘ungulates’ GMM returns 14 RWs. For the convergence analyses we only the first five RWs that explain cumulatively 89.69% of variance.

|  |  |  |  |
| --- | --- | --- | --- |
|  | **eigenvalues** | **%Variance** | **%Cumulative** |
| **RW1** | **8.59E-03** | **45.19** | **45.19** |
| **RW2** | **3.22E-03** | **16.93** | **62.11** |
| **RW3** | **2.14E-03** | **11.23** | **73.34** |
| **RW4** | **1.77E-03** | **9.30** | **82.65** |
| **RW5** | **1.34E-03** | **7.04** | **89.69** |

**Supplementary Table S2- RWA results for ‘Ungulatomorpha’. Eigenvalues: eigenvalues of the Covariance matrix; %Variance: variance for each RW; %Cumulative: cumulative variance.**

**Imposing ancestral states**

We tested the effect of imposing ancestral states phenotypes in recognizing patterns of convergence. To this aim, we ran *search.conv* on the felids data with the automatic mode but indicating the ancestor of all barbourofelids mandible to have the same phenotype (PC scores in this case) of the highly derived *Barbourofelis fricki*. The most common ancestor of all machairodonts was similarly set to have the same phenotype of the highly derived machairodont *Smilodon fatalis*. We found the recognized convergence patterns are the same regardless of whether the ancestral phenotypes to all barbourofelids and machairodont are specified or fitted either (Supplementary Figure S3).


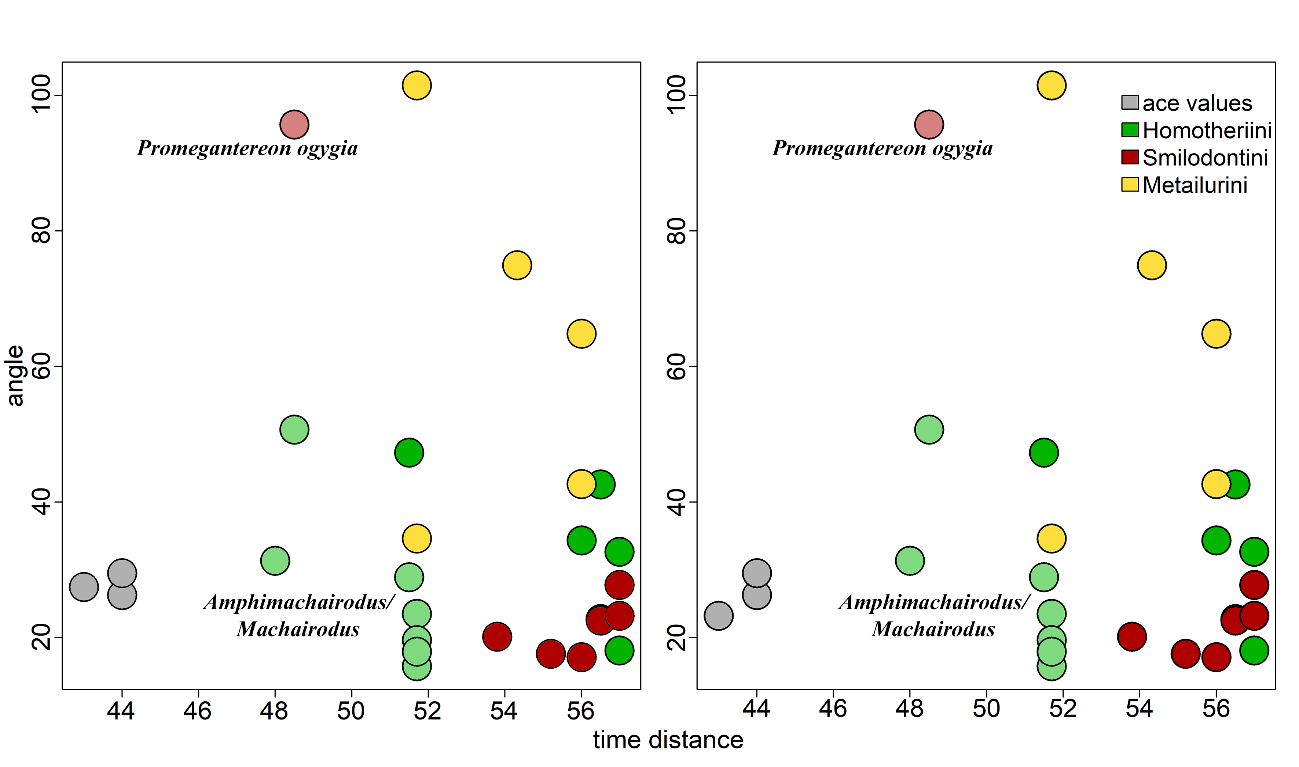


**Supplementary Figure S3- Angles between species and ancestral phenotypes (gray dots) obtained either using fitted (left) or imposed (right) ancestral states. Red dots, convergent (on barbourofelids) smilodontini. Light red, non-convergent (on barbourofelids) smilodontini. Green, convergent (on barbourofelids) homotheriini. Light green, non-convergent (on barbourofelids) homotheriini. Yellow, metailurini.**

**Single state**

**
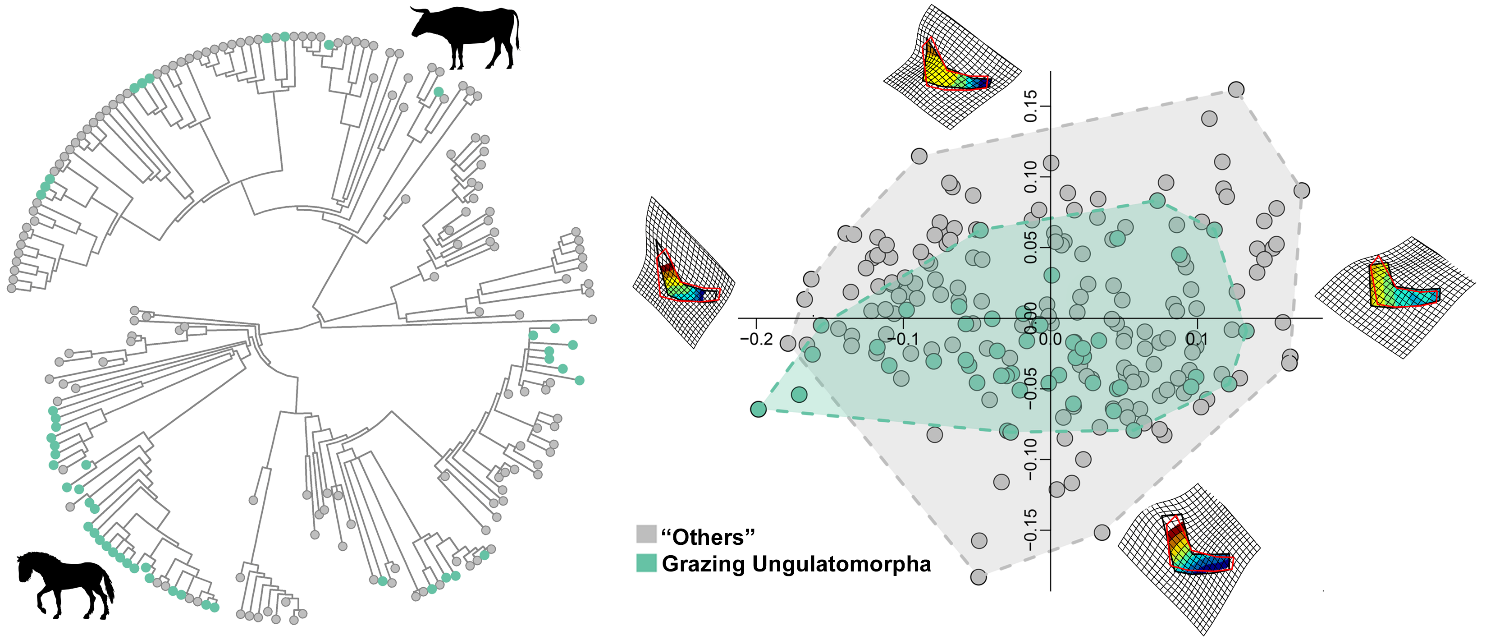
**

**Supplementary Figure S4 -The distribution of individual species per state (gray = background state (others), green = grazing Ungulatomorpha) on the ‘ungulate’ tree (left). To the right, the PC1/PC2 plot showing the position of the convergent state (grazers) compared to the rest of the tree. Deformation grids are shown at the extremes of both axes. Animal silhouettes were available under Public Domain license at phylopic (**[**http://phylopic.org/**](http://phylopic.org/)**). Specifically, *Bos primigenius* (http://phylopic.org/image/dc5c561e-e030-444d-ba22-3d427b60e58a/) image by DFoidl (modified by T. Michael Keesey) and *Equus ferus* (http://phylopic.org/image/85d95128-912c-427a-9542-138e1dbf5651/) image by Mercedes Yrayzoz (vectorized by T. Michael Keesey) are available for reuse under the Creative Commons Attribution 3.0 Unported (**[**https://creativecommons.org/licenses/by-sa/3.0/**](https://creativecommons.org/licenses/by-sa/3.0/)**).**

**Phylogenetic Principal Component Analysis**

The angle theta represents the correlation coefficient between the two phenotypic vectors. There are other phylogenetic comparative tools developed to deal with the phylogenetic correction of the correlation between phenotypes, most notably phylogenetic PCA (Revell 2009; Polly et al. 2013). We performed phylogenetic PCA by using the function phyl.pca in the R package phytools (Revell 2012) taking into account phylogenetic effects to correct the data for nonindependence (Revell 2009). Phylogenetic PCA was implemented using both the Felid and “Ungulatomorpha” data to compare this ordination technique with our results (Supplementary Figure 5).


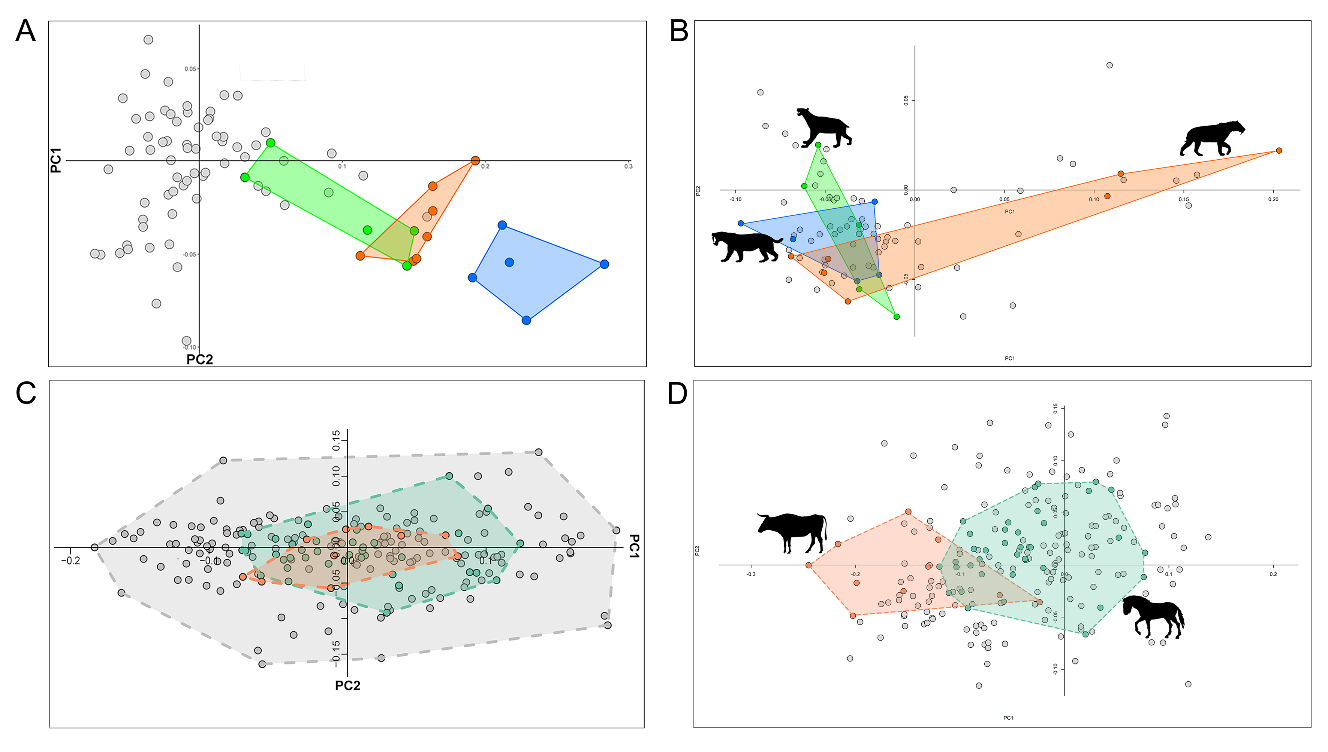


**Supplementary Figure S5 – A,C) PC1/PC2 plot in Figures 5 and 6 in the main manuscript, obtained by applying search.conv. B) phylogenetic PCA plot showing the position of the sabertooth clades. D) phylogenetic PCA plot showing the position of the “Ungulatomorpha” grazers. Plots in B and D were obtained by applying phylogenetic PCA. Animal silhouettes were available under Public Domain license at phylopic (**[**http://phylopic.org/**](http://phylopic.org/)**). The silhouette for *Homotherium* was available for reuse under the Creative Commons Attribution 3.0 Unported (https://creativecommons.org/licenses/by-sa/3.0/) at http://phylopic.org/image/c6c2d17b-56b3-4c87-97c4-cd2b7de365fa/ (image by Zimices). The silhouettes for *Smilodon* and *Barbourofelis* are our own work. *Bos primigenius* (http://phylopic.org/image/dc5c561e-e030-444d-ba22-3d427b60e58a/) image by DFoidl (modified by T. Michael Keesey) and *Equus ferus* (http://phylopic.org/image/85d95128-912c-427a-9542-138e1dbf5651/) image by Mercedes Yrayzoz (vectorized by T. Michael Keesey) are available for reuse under the Creative Commons Attribution 3.0 Unported (**[**https://creativecommons.org/licenses/by-sa/3.0/**](https://creativecommons.org/licenses/by-sa/3.0/)**).**

**Simulation sets using the time (istead of number of nodes) to measure the distance between clades**

We produced 1000 paleontological phylogenies with at least 80 species, by using the function *sim.bdtree* (package geiger) with birth and death rates set at 0.5 and 0.2, respectively. In order to skew the distribution of node heights, we rescaled the trees by applying the Pagel’s delta transformation setting delta at either 0.5 (for 500 trees) or 2 (for 500 trees). For each phylogeny, we generated a multivariate phenotype ***y*** according to the Brownian Motion model of evolution by using the function *fastBM* in the package ape. Then, we selected two unrelated clades ***s*** and ***s’*** to be convergent. We picked two clades within the tree having as many as one twentieth to one quarter of the tree tips and being distant from the root no more than 80% of the tree height. For each of them, we simulated an OU model of phenotypic evolution. The optimal value *theta* was obtained by multiplying the maximum of each ***y*** variable by a random value (***f***) sampled between 0.5 and 2. The strength parameter *alpha* was randomly sampled between 1 and 50. We replaced the original ***y*** values of ***s*** and ***s’*** with the new OU phenotypes. Finally, we performed *search.conv* on each set of tree and data, by setting the time distance between ***s*** and ***s’*** minus one as minimum distance between the clades to be scanned (*min.dist*). The results indicated that the Type I error rate (finding convergence when the phenotype is simulated according to BM throughout the tree) is 2% (20 errors, 11 with delta set at 2, 9 with delta set at 0.5). The Type II error rate (failure to find convergence) is 5.2% (52 errors, 24 with delta set at 2, 28 with delta set at 0.5). The R code illustrating this procedure and used to assess Type I and II error rates is available within Supplementary File S4.


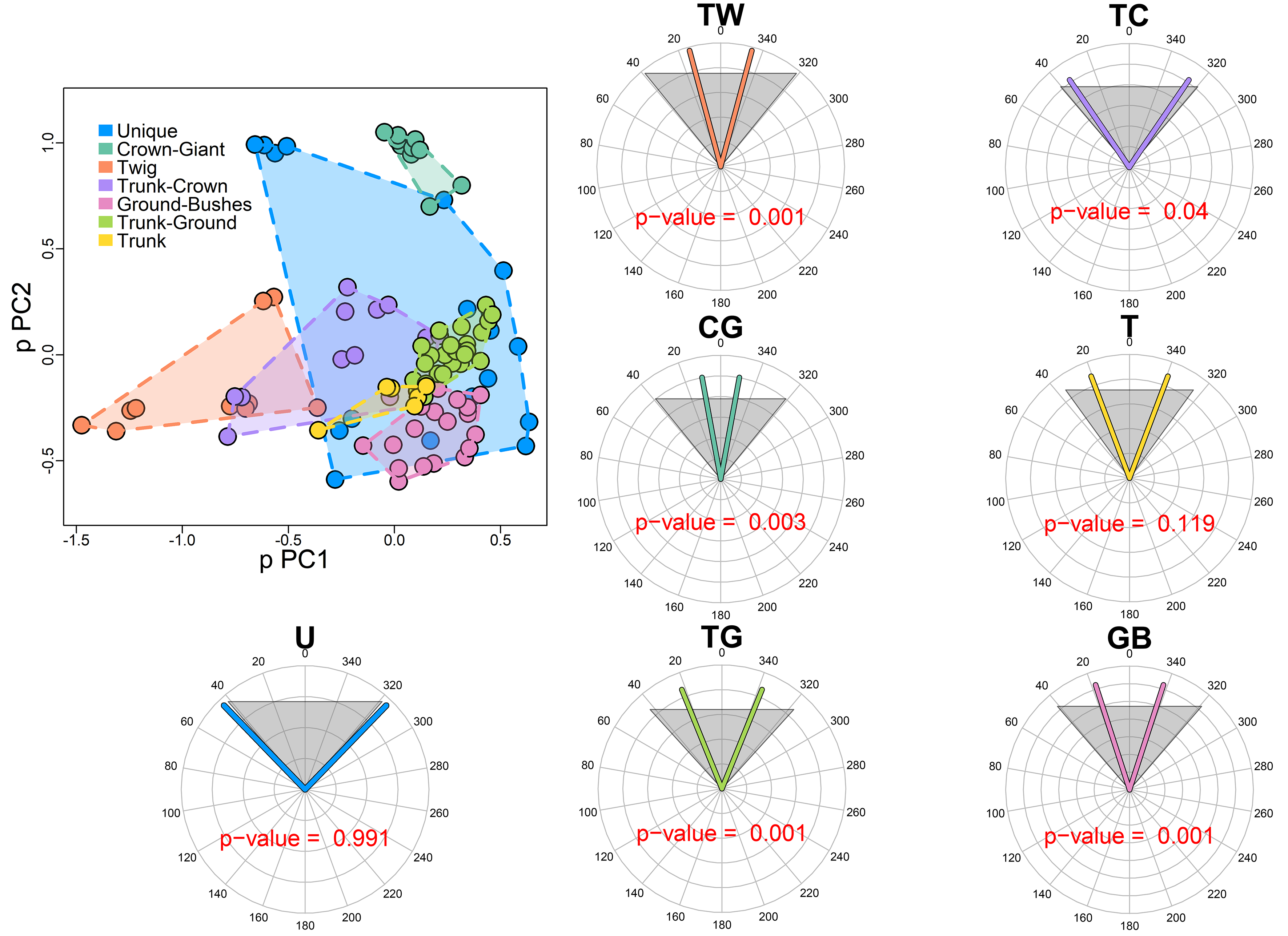


**Supplementary Figure S6 – Convergence within *Anolis* ecomorphs. Individual ecomorphs are indicated in the pPCA (phylogenetic PCA scores) space to the upper left. The polar plots represent the mean angle formed by pair of tips per ecomorph (color lines) superimposed on the 95% confidence interval of the random angle (grey shaded area). The angles are not divided by the mean distance between the tip pairs direct ancestors, yet the significance value (in red) takes the temporal distance into account.**

**References**

Paradis E., Claude J. & Strimmer K. (2004). APE: analyses of phylogenetics and evolution in R language. Bioinformatics 20: 289-290.

Raia, P., Carotenuto, F., Meloro, C., Piras, P., & Pushkina, D. (2010). The shape of contention: adaptation, history, and contingency in ungulate mandibles. *Evolution*, *64*(5), 1489-1503.

Piras, P., Silvestro, D., Carotenuto, F., Castiglione, S., Kotsakis, A., Maiorino, L., & Vero, V. A. (2018). Evolution of the sabertooth mandible: A deadly ecomorphological specialization. *Palaeogeography, Palaeoclimatology, Palaeoecology*, *496*, 166-174.

Polly, P. D., Lawing, A. M., Fabre, A.-C., & Goswami, A. (2013). Phylogenetic Principal Components Analysis and Geometric Morphometrics. Hystrix, the Italian Journal of Mammalogy, 24(1), 33–41.

Raia P., Castiglione S., Serio C., Mondanaro A., Melchionna M., Di Febbraro M., Profico A., Carotenuto F. (2018). Package RRphylo v2.2.0 <https://github.com/pasraia/RRphylo>

Revell, L. J. (2009). Size‐correction and principal components for interspecific comparative studies. Evolution: International Journal of Organic Evolution, 63(12), 3258-3268.

Revell LJ. (2012) phytools: an R package for phylogenetic comparative biology (and other things). Met Ecol Evol., 3: 217–223.
